# Human Alveolar Echinococcosis in Poland: 1990–2011

**DOI:** 10.1371/journal.pntd.0001986

**Published:** 2013-01-03

**Authors:** Wacław L. Nahorski, Józef P. Knap, Zbigniew S. Pawłowski, Marek Krawczyk, Jerzy Polański, Jerzy Stefaniak, Waldemar Patkowski, Beata Szostakowska, Halina Pietkiewicz, Anna Grzeszczuk, Iwona Felczak-Korzybska, Elżbieta Gołąb, Natalia Wnukowska, Małgorzata Paul, Elżbieta Kacprzak, Elżbieta Sokolewicz-Bobrowska, Jolanta Niścigorska-Olsen, Aleksandra Czyrznikowska, Lidia Chomicz, Danuta Cielecka, Przemysław Myjak

**Affiliations:** 1 Department of Tropical and Parasitic Diseases, Chair of Tropical Medicine and Parasitology, Institute of Maritime and Tropical Medicine, Medical University of Gdańsk, Gdynia, Poland; 2 Department of Epidemiology, Medical University of Warsaw, Warszawa, Poland; 3 Department and Clinic of Tropical and Parasitic Diseases, Medical University of Poznań, Poznań, Poland; 4 Chair and Department of General, Transplant and Liver Surgery, Medical University of Warsaw, Warszawa, Poland; 5 II Chair and Department of General and Vascular and Oncological Surgery, Medical University of Warsaw, Warszawa, Poland; 6 Department of Tropical Parasitology, Chair of Tropical Medicine and Parasitology, Institute of Maritime and Tropical Medicine, Medical University of Gdańsk, Gdynia, Poland; 7 Department of Infectious Diseases and Hepatology, Medical University of Białystok, Białystok, Poland; 8 University Centre of Maritime and Tropical Medicine in Gdynia, Gdynia, Poland; 9 Department of Parasitology, National Institute of Hygiene, Warsaw, Warszawa, Poland; 10 Department of Infectious Diseases, Voivodeship Hospital in Szczecin, Szczecin, Poland; 11 Chief Sanitary Inspectorate, Warsaw, Warszawa, Poland; 12 Department of Medical Biology, Medical University of Warsaw, Warszawa, Poland; 13 Department of General Biology and Parasitology, Medical University of Warsaw, Warszawa, Poland; Queensland Institute of Medical Research, Australia

## Abstract

**Background:**

Alveolar echinococcosis (AE) caused by *Echinococcus multilocularis* infections is a dangerous old disease in the Northern Hemisphere. The aim of the paper was to collect and analyze data on human AE in Poland in the last two decades.

**Methodology/Principal Findings:**

The sources of data were both the cases officially registered and detected by an active field and laboratory surveillance. The cases were verified by clinical, epidemiological, and laboratory criteria. Altogether 121 human cases of AE were detected. Among these 83 (68,6%) cases were classified as confirmed, 16 as probable and 22 as possible. During the two decades a continuous increase in detection rate was noticed. The cases were 6–82 years old at the time of diagnosis (mean - 47.7 years). Sex ratio M/F was 0.86/1.0. The AE was fatal in 23 (19%) patients (mean age at death - 54.1 years). Family agglomeration of AE was found in 4 foci, involving 9 patients. Seventy six of the cases were diagnosed in an advanced stage of disease. In all cases the liver was the primary location of AE. In 30 (24.8%) patients a spread to other organs was observed. Ninety four of the patients were treated with albendazole. In 73 (60%) patients a surgical operation was performed, including 15 liver transplantations.

**Conclusions/Significance:**

The studies confirmed that AE is an emerging disease in Poland, which is the fourth country in Europe with over 120 cases detected. The results also indicate the need of a wider national programme for implementation of screening in the highest AE risk areas (north-eastern Poland) with an effort to increase the public awareness of the possibility of contracting *E. multilocularis*, and above all, training of the primary care physicians in the recognition of the risk of AE to allow for an early detection of this dangerous disease.

## Author Summary

Human alveolar echinococcosis (AE) is a consequence of accidental ingestion of the eggs of the tapeworm *Echinococcus multilocularis*. AE constitutes a serious and increasing medical and epidemiological problem in many countries including Poland. The natural environments with a high AE prevalence are the areas where the definitive hosts (foxes, raccoons) and the intermediate hosts (rodents) live. The increasing population of infected foxes that penetrate nearby the village and urban buildings and infected dogs poses a certain risk to human health. This report is a joint clinical and epidemiological analysis of 121 AE cases recorded in the years 1990–2011 in Poland. The highest percentage of *E. multilocularis* infected foxes in the north-eastern regions of Poland (about 40% of the examined population infected) bears a clear-cut relation to the highest incidence of detected AE human cases in those areas. An epidemiological and clinical survey of the local population conducted for over a dozen years has contributed to the discovery of several new AE cases. Alveolar echinococcosis in humans caused by *Echinococcus multilocularis* infection belongs to the emerging diseases in Poland where a continuous increase in the detection rate was noticed during the last two decades.

## Introduction

During the last three decades there has been a continuous increase in worldwide interest both in human and animal alveolar echinococcosis (AE), mainly due to a substantial increase in the number of recorded AE human cases.

Human alveolar echinococcosis is caused by the larval stage of *Echinococcus multilocularis*, occurring in at least 42 countries of the northern hemisphere. Recent studies in Europe and Asia have shown that the endemic area of *E. multilocularis* is larger than previously suspected and an invasion among foxes has regionally expanded from rural to urban areas [1]. Alveolar echinococcosis is old disease in Europe [2]. In the last half-century, the most frequent cases of human AE in Europe were described in France, Germany, Switzerland and Austria [1], [3], and more recently, also in Lithuania, the nearest neighbouring country, where 96 human AE cases were recorded in the years 1997–2008 [4]–[5]. Recently the most numerous casus of AE in humans have been reported from China [6]–[8].

The clinical progress of human AE is very slow: the period between exposure and the appearance of symptoms may be as long as several to dozen or more years [9]–[10]. The increase in the incidence rate of human AE in Poland [11] in the recent decade is particularly worrying. Human AE is diagnosed mainly in areas with a high prevalence of infected red foxes, a fact testifying to an alleged link between the occurrence of *E. multilocularis* in animals and human infections in the locally community [12]–[14].

In Poland the fox population increased from 67 000 in 1995 to 220 000 in 2006 [13]. The average *E. multilocularis* prevalence in the red fox in Poland is 2.6%, the highest infection rate being in the Varmia – Masuria Province (mean 39.6%, in some districts varies from 50.0–62.9%), in the Sub – Carpathian Province (36.8%) and in the Province of Pomerania (mean 7.9%) [13].

In Poland by 1980 human AE had been known from the two individual case reports [15]–[16]; and two other cases in 1984 and 1988 were diagnosed retrospectively [17]. However in the years 1990–2011 a significant increase in morbidity including fatal cases was observed [11]. Several cases were individually reported [17]–[29]. The EurEchinoReg includes 14 cases notified [1], two out of which were recognized in the eighties, 6 in the years 1990–1995 and another 6 in the years 1996–2000.

In the first half of the nineties the Poznań Clinical Centre in cooperation with the WHO Informal Group on Research in Echinococcosis made an attempt to coordinate scientific research into AE in 4 clinical centers in Poland (Białystok, Gdynia, Poznań, Warszawa) [30]. Four scientific conferences were held (Poznań 1996, 2002, 2004 and Warsaw 1999). National Reference Laboratories for serologic AE differentiation were organized [31]. Based on the information from five clinical centers the first attempt was made in 1998 to register the AE cases using the EurEchinoReg programme [1]. In 2003 in an effort to formalize and extend the register, the Chief Sanitary Inspectorate (CSI) of Poland was asked to continue the National AE Register. Preliminary data of CSI were presented during CSI Conference in Warsaw, 2006 (unpublished).

In the treatment of AE in humans chemotherapy (mainly with albendazole (ABZ)) and radical surgery are used. In Poland treatment with albendazole meets difficulties because the high cost of the drug.

We aim to analyse the clinical and epidemiological data on 121 autochthonous human AE cases registered by CSI in Poland in the years 1990–2011, collected by retrospective and prospective analysis and as well as an active epidemiological field surveys. The results of this analysis were partly reported at conferences in 2010 [11].

## Materials and Methods

### Identification and definition of AE human cases

The studies were conducted in areas with a high prevalence of *E. multilocularis* in foxes. Retrospective, current and prospective analysis of persons, was done by using clinical, laboratory and field data. The most numerous group of patients emerged from the persons who presented at the doctor's with abdominal pain (n = 94). An additional 27 cases were detected (n = 16) or suspected cases confirmed (n = 11) during field studies carried out in the years 1997–2008, when a total of 7308 serum samples were collected from persons living in areas with high prevalence of *E.multilocularis* in foxes. The respective cases were categorized based on the criteria described by Brunetti et al. [10]. The diagnosed cases were defined as: confirmed, probable or possible.

### Laboratory investigations

The tissue specimens taken during liver biopsy and/or surgery were sent for histopathology, ultrastructural and molecular studies to confirm the diagnosis. The histological preparations were stained with haematoxylin and eosin and by the Periodic acid-Schiff (PAS) technique [10]. Such techniques as trichrom and Azan-staining, as well as ultrastructural investigations by transmission electron microscope were also included [32]. Polymerase Chain Reaction (PCR) was performed according to the procedure described by Dinkel et al. [33] and Myjak et al. [24]. The following serological tests were used: indirect haemagglutination test (IHA) (Echinoccocose_HAkit, bioMėrieux, France), Enzyme linked immunosorbent assay (ELISA)-IgG (Immunodiagnostica Gmbh, Germany) or ELISA-IgG (Bordier Affinity Products S.A. Crissier, Switzerland) detects IgG antibodies to *Echinococcus* spp; ELISA Em2plus (Bordier Affinity Products S.A.) – which detects antibodies to *E. multilocularis* and as a confirmation test immunoblotting (Western-blot) (LDBIO, France), which helps to differentiate between *E. granulosus* and *E. multilocularis* in 70% cases. All tests were applied and read according to the manufacturer's instructions.

Serological tests were carried out mostly in 3 scientific diagnostic laboratories: Department of Tropical Parasitology Medical University of Gdańsk (DTP MUG) (59 cases, 48,8%–384 serum samples), Department of Parasitology National Institute of Hygiene (DP NIH) (33 subjects, 27.3%–39 serum samples) and Department and Clinic of Tropical and Parasitic Diseases Medical Uniwersity of Poznań (DCTPD MUP) (17 cases, 14%–49 serum samples). Molecular studies were performed in DTP MUG (20 cases, 16.5%) and ultrastructural investigations in the Department of Medical Biology Medical University of Warsaw (DMB MUW) (9 patients, 7.4%). The histopathological examinations were conducted in local hospitals where the subjects were treated.

### Clinical investigations

Most AE affected patients - 95 (78%) were hospitalized in the specialised clinical centers: Department of Tropical and Parasitic Diseases Medical University of Gdańsk (DTPD MUG) (52 patients, 43%), Department of Infectious Diseases and Hepatology Medical University of Białystok (DIDH MUB (16 patients,13.2%), DCTPD MUP (16 subjects, 13.2%), Department of Infectious Diseases of Voivodeship Hospital in Szczecin (5 cases, 4.1%) as well as in the surgical divisions: Chair and Department of General, Transplant & Liver Surgery, Medical University of Warsaw (CDGTLS MUW) (29 patients, 24%, including 12 liver transplantations and other major surgical procedures) and II Chair and Department of General & Vascular and Oncological Surgery Medical University of Warsaw (CDGVOS MUW) (12 patients, 9.9%, including 9 cases of major surgery). The remainder (n = 26, 21.5%) were hospitalized in nonacademic hospitals. Some individuals were treated in two or three clinical centers.

When possible (mostly in CDGTLS MUW, CDGVOS MUW and DCTPD MUP) the diagnosed 34 AE cases were assigned to one of the clinical Parasitic-Neighbouring-Metastasis (PNM) coded groups [34]. PNM grading is based on staging of neoplasm and determines appropriate treatment decisions, the duration of chemotherapy and may also provide prognostic information.

The written consent of the Local Ethics Commission of the Medical University of Gdańsk was obtained to perform the epidemiological and clinical field studies (routine clinical diagnostic procedures being carried out during hospitalization). We obtained a written consent from every patient involved.

## Results

### Diagnostic data

In 1990–2011 a total of 121 new AE cases (incidence 0.014) have been diagnosed, including 27 (22.3%) cases found during field studies in endemic areas screened for human AE. These 121 cases of alveolar echinococcosis include also 12 cases diagnosed in the nineties and reported to the EurEchinoReg [1].

Suspicion of AE was mainly based on ultrasonography (US) examination of the liver. This finding was usually followed by serologic tests, in conjunction with clinical and imaging studies, the results of which were regarded as an indication to treatment with albendazole or surgery.

Serologic tests were performed in 114 subjects (94.2%). In 4 patients in the initial stage of the investigation the above tests were not done, whereas information on diagnostic issues was missing in three patients. Immunoblotting (Western-blot) was used to study 106 serum samples (87.6% patients), ELISA Em2plus in 104 cases (86%), ELISA–IgG was performed in 84 cases (69.4%), whereas IHA in 17 (14%). Histopathological examinations were carried out in 84 (69.4%) patients, ultrastructural studies in 9 (7.4%) subjects and molecular studies (PCR) in 20 (16.5%).

Out of the 121 cases recorded, 83 (68.6%) were classified as confirmed, 16 (13.2%) as probable and 22 (18.2%) were classified into the “possible” category according to the Brunetti et al. criteria [10]. In some individual cases it was difficult to fulfill all the diagnostic criteria. As confirmed AE cases were also regarded those positive according to the clinical, epidemiological and imaging criteria, but were negative serologically (4 cases) or the serological tests were not performed (4 cases), and or a specific test (ELISA–Em2plus or western blot) turned out to be positive, but another specific test was negative (5 cases) or was not performed (10 cases).

According to the accepted criteria [10], 32 (26.4%) patients, in whom either histopathological or molecular studies were not done but the serological tests were performed, were classified into the “probable” or “possible” categories in spite of the fact that the epidemiological, clinical and imaging criteria were in favour of AE.

### Epidemiological data

**Figure 1:**
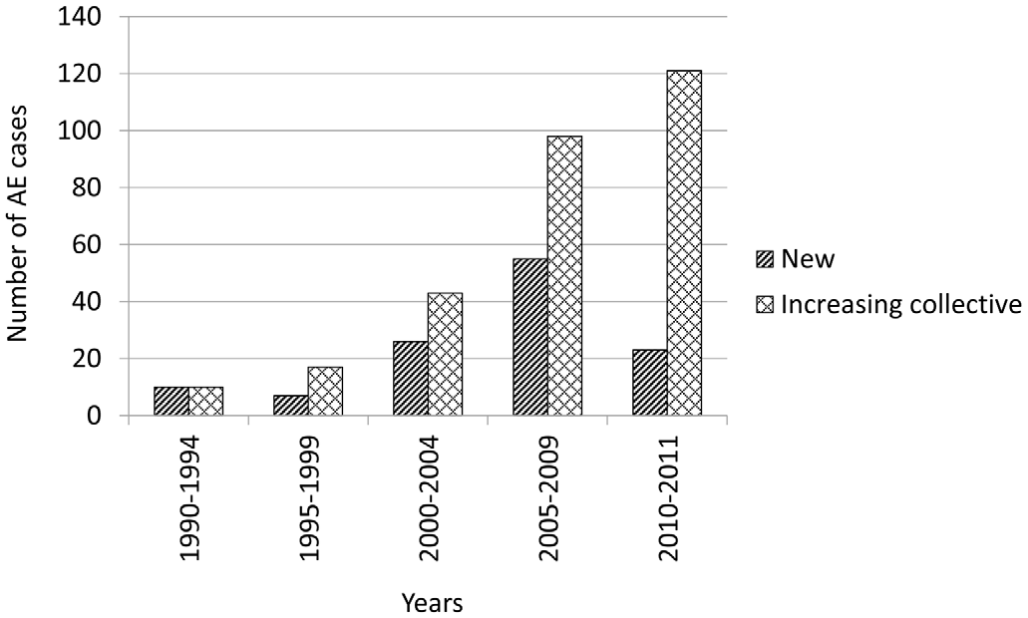
Human AE cases in Poland diagnosed in 5-year interval.

In successive 5 year periods under investigation (1990–2011) there was a steady increase in the detection of AE cases in Poland, the most significant rise being observed in the years 2005–2009 (55 cases, 45.5%), however over 23 months of 2010–2011 as many as 23 new AE cases have been diagnosed (Fig. 1).

Among the 121 patients recorded, 65 (53.7%) were females and 56 (46.3%) males (M: F ratio – 0.86/1.0).

**Figure 2:**
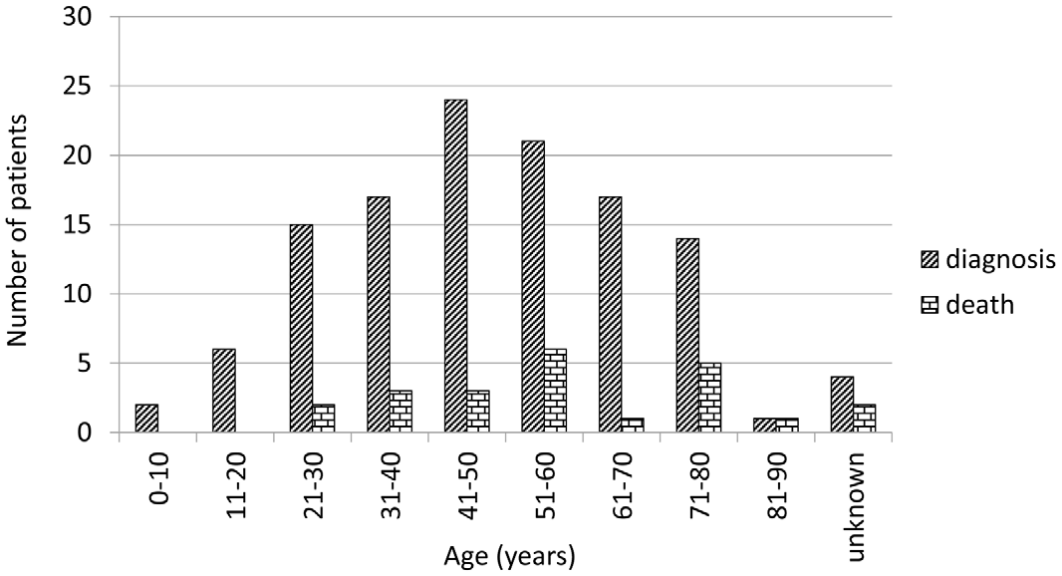
Age of 117 patients in the year of AE diagnosis and death of 21 persons.

Age at the diagnosis of 118 persons infected with AE ranged from 6 to 82 years, (mean age 47.6). The age of 3 women and one man could not be determined. Specifically, in 62 women the age varied from 6 to 82 years (mean 49.2 years), in 55 men from 10 to 77 years (mean 45.7 years) (Table 1). AE was most commonly diagnosed in the age group 41 to 50 years (Fig. 2).

**Table 1:** Sex and age of 117 patients in the year of AE diagnosis and death of 21 persons.

Sex	Diagnosis			Death		
	N	Age (years)		N	Age (years)	
		Range	Mean		Range	Mean
Female	62^a^	6–82	49.2	11^b^	24–87	57.2
Male	55^a^	10–78	45.7	10^b^	27–76	48.8
Total	117	6–82	47.6	21	24–87	53.2

a In three women and one man their age could not be determined,

b In one women and one man their age could not be determined.

During the observation period 23 persons died (19%) aged from 24 to 87 years, an average of 50.8, the highest death rate was in 51 to 60 years (Fig. 2). The immediate cause was: the progress of the AE or its complications, and in five cases, another concomitant disease (e.g. heart failure). The average survival time after diagnosis was 4.54 years, ranging from 2 months to 15 years.

Four of the cases were children aged 6, 10, 11 and 11 years, which indicates a very early exposure to *E. multilocularis*. All four children live in a rural area. Their illness was diagnosed in the years 2005–2008, in one case as confirmed and in the three as possible. Two children were treated with albendazole and, in addition to oral therapy, one of them underwent surgical removal of the hepatic lesion. Unfortunately, no insight into the patients' files of the remaining two children was not possible.

**Figure 3:**
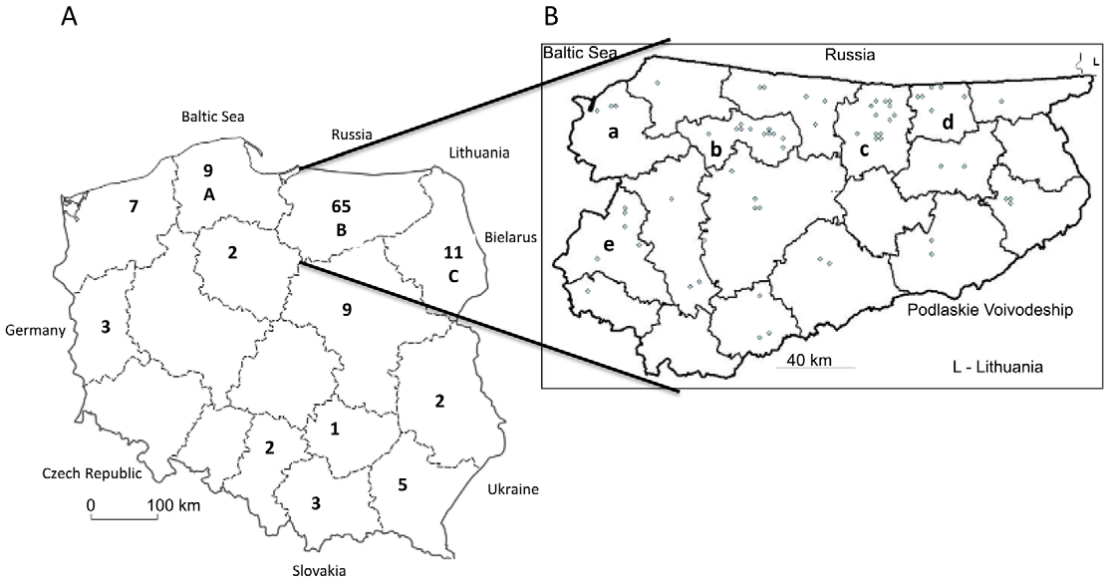
Geographical distribution of human AE cases in Poland. **A – Number of AE cases in each province.** Localizations of three patients could not be determined. A – Pomorskie Province; B – Varmia-Masuria Province; C- Podlaskie Province. **B – Distribution of 65 AE cases in the Varmia - Masuria Province.** Global area amounts to 24 173 km^2^, and population 1 426 155 (2008). a – Elbląski district; b – Lidzbarski district; c – Kętrzyński district; d – Węgorzewski district; e – Iławski district.

One hundred eighteen cases of AE in humans in Poland were observed in 12 of 16 provinces (in 54 of total 379 districts -14%). These cases were distributed unevenly, the largest number (65 cases, 53.7%) was recorded in the Varmia and Masuria Province (Fig. 3 A, B) and Podlaskie (11 cases) (north eastern Poland) (Fig. 3 A). The origin of three patients could not be determined.

The highest incidence of detected AE cases was in the Varmia – Masuria Province (Table 2). In this Province, the highest average yearly incidence (over 2/100 000) of recorded AE cases occurs in 4 communities: Srokowo, Barciany, Budry and Kiwity located in three districts. Furthermore in the two other communities: Młynary and Zalewo the incidence was 1.95–1.97.

**Table 2:** Incidence of alveolar echinococcosis in Varmia-Masuria province, Poland, during 1990 through 2011.

Geographic location		N^a^	Detection rate^b^
Poland		121	0.014
Varmia-Masuria Province		65	0.20
**Districts**	**Communities**		
Kętrzyński		13	0.89
	Srokowo	5	**5.3**
	Barciany	3	**2.02**
Węgorzewski		6	1.16
	Budry	3	**3.9**
Lidzbarski		11	1.15
	Kiwity	2	**2.59**
Elbląski		3	0.07
	Młynary	2	1.97
Iławski		5	0.25
	Zalewo	3	1.95
Other districts		27	0.13

a Total number of cases (1990–2011),

b Per 100 000 population (yearly - mean).

Family aggregation of the 9 cases (7.4%) was observed in 4 focuses. In one of them the afflicted persons were: a mother and two daughters, in another one a brother and a sister [21], in the third focus two brothers, in the fourth focus: mother and son. Out of these patients only 3 are still alive. The 3 focuses were identified in areas of a high prevalence of *E. multilocularis* in foxes.

An overwhelming majority of the patients (73.1%) were living in rural areas and had a long – lasting occupational or/and environmental contact with the forest (going bilberry picking and mushrooming. As many as 87 (72%) of the patients had its own household dog.

### Clinical data

In all 121 cases, the liver was the primary location of AE. In most cases – 81 (74%) liver was the only affected organ, but in 30 (25.8%) patients there was a spread to other organs either in the form of metastatic dissemination (M – metastases) or infiltration of adjacent structures (mainly the bile ducts).

Metastases were found in the lung – 11 cases, the brain – 4 cases, the peritoneum – 3 cases, multiorgan dissemination – 4 cases, metastatic involvement of the ovary was evidenced in 2 patients, of the lymph nodes – 3 cases, bony metastasis to the crus tibia – 1 case, metastatic involvement of the prostate and gallbladder – 1 case of each location; infiltration of the liver vessels and adjacent internal organs was recognized in 6 cases. Liver failure occurred in 6 patients, renal failure imposing the necessity of chronic dialysis in 3 subjects.

Chemotherapy with albendazole was instituted in at least 94 (77.7%) patients including the “during and after surgery” cases. There was some irregularity in taking albendazole due to its price and availability as well as difficulties of rural patients in contacting the specialized medical centres.

Toxic effects of albendazole were noted at least in 8 (6.6%) patients taking the forms of *hepatitis* (n = 4), *leucopenia* (n = 4), *alopecia* (n = 2). Consequently, in four cases it led to ABZ treatment interruption and to initiation of the treatment with nitazoksanide (“Alinia”). The treatment has not been continued because of lack of registration of the drug and its high cost. In patients treated with Alinia no signs of liver damage have been observed.

AE – related surgery, sometimes repeated was performed in 73 (60.3%) patients. Out of the above subjects, 15 patients underwent liver transplantation. In 43 patients surgery consisted in hepatic lobectomy, segmentectomy or excision of the local parasitic lesion. In 15 patients diagnostic laparotomy was undertaken, followed by no further surgery. 26 subjects were spared from any surgical procedure. Detailed date on treatment were absent in 22 patients.

In the clinic CDGTLS MUW, 29 patients were hospitalized, of whom 26 patients were operated because of AE (8 women and 18 men). In the surgically treated group, there were 12 liver transplantations, 11 liver resections with the intention of the radical operation and three explorative or diagnostic laparotomies. In these 26 patients, clinical severity assessed according to PNM classification was as follows: Stage I - 4 patients, stage II - 3 patients, stage IIIa - 2 patients, stage III B - 9 patients, and stage IV - 8 patients. The wide range of operations in individual patients, was as follows: 12 patients – orthotopic liver transplantation (OLT), 6 patients – right hemihepatectomy, one patient – right hemihepatectomy extended to segment IV, 1 patient - central liver resection, 2 – left hemihepatectomy, 1 – left lateral bisegmentectomy.

In 13 patients there were no complications, in 2 patients biliary fistula was formed, there was a case of pleural effusion, pneumonia in one, in 4 postoperative cases wound infections developed pulmonary embolism occurred in one and two patients died. One patient died after right hemihepatectomy extended to segment IV. The second patient died after 1.5 years of successful transplantation, due to necrosis of the small intestine in the course of the gastrointestinal obstruction.

In the clinic CDGVOS MUW, 12 AE patients have been hospitalized, while 9 subjects (5 women and 4 men) underwent major hepatic surgery. The PNM grading was as follows: stage IIIA – 1 patient, IIIB – 3 patients, stage IV – 5 subjects. Major hepatic resections were usually accompanied by additional surgical procedures (duodenal surgery, right lung tumorectomy, partial resection of the diaphragm, operation on the common bile duct, and operation on the ileum and adjacent iliac muscles, partial excision of the portal vein and inferior vena cava, as well as an external drainage of the cyst cavity). The weight of the removed parasite containing tissues varied from 498 to 2470 grams (the average 1214 grams). The duration of follow – up after operation varied from 10 months to 16 years [35].

In total 35 patients underwent surgery in the reference specialised surgical clinics. Other surgical procedures (including three OLT) in 38 AE patients were performed in different surgical wards in Poland.

## Discussion

In the period of investigations (1990–2011) a steady increase in the incidence of AE cases in Poland was noted (Fig. 1). A similar tendency was also observed in Germany [1] and in Lithuania [4]–[5], whereas in France [1], [3] and in Austria [1] the incidence rate remained at a constant level.

The presented group of 121 autochthonous human AE cases sets Poland in the rank of other European countries (France, Switzerland, Germany and Poland) with the number of recorded AE cases exceeding 120.

Human AE cases were found in 12 among 16 Provinces in Poland. Their geographical distribution was not equal. The highest detection was observed in the Varmia – Masuria and Podlasie Provinces located in the north – eastern part of Poland) (Fig. 3 A, B). These Provinces are very close to Lithuania (surface: 65 300 km^2^, 3.4 million inhabitants), where between 1997 and July 2006 as many as 80 human AE cases were diagnosed [4], and rose up to 96 by July 2008 [5]. These facts are suggestive of a certain underestimation of the incidence of human AE cases in the above region. The real number of human AE cases in Poland is presumably much higher. Real number of the unreported cases may be caused by: the non-diagnosed AE cases (probably due to diagnostic difficulties), misdiagnosed usually as primary or secondary malignancies of the liver, the cases with imprecise diagnosis “Echinococcus – caused disease”, as well as true AE cases remained unreported to State Epidemiological Sanitary Inspection, despite of the fact that a legal obligation to report all AE cases is in force.

Although the red fox is the definitive host of *E. multilocularis*, the epidemiological data suggest that also dogs and cats play a more and more important role in the transmission of the parasite onto man [36]–[38]. Some authors believe, these animals pose a major threat to man [7].

In 2003, prof. Pawłowski, who [39] initiated a program of modern diagnostics and registration of AE patients in Poland and coordinated it with the European operations [1] has decided to pass the further studies and registration to CSI.

This report, based on a planned multi-center clinical study, as well as field studies is the up-dated collection of data on all human AE cases diagnosed and treated in Poland.

The rise in the number of established human AE cases in Poland is allegedly due to several factors: it may be associated with an increase in red fox population causing an increased risk of infection [14], but also it may be a result of the introduction of imaging techniques and modern diagnostic methods (histochemical, ultrastructural, serologic and molecular) to general diagnostic practice. Also increased public and medical practitioners' awareness of the possibility AE can play a part in early and effective disease detection. It should be kept in mind that wild fruits and mushrooms as well as garden fruits and vegetables can not be ruled out as a source of infection. Recently we detected the DNA of *E.multilocularis* in 13% of samples taken from fruits and vegetables (Szostakowska et al. 2012). The role of the dog and cat in AE transmission cannot be clarified at present through lack of the relevant studies in Poland.

In DTP MUG [24], DCTPD UMP [26] and DP NIH [40] a routine serological diagnosis of AE based on 3 commercial tests was introduced in the nineties, and since 2000 molecular diagnosis of AE has been employed in DTP MUG [24]. The Poznań Clinical (DCTPD UMP) instituted the serological studies involving the adhesive particles aimed at evaluating the clinical course and the degree of advancement of alveolar echinococcosis of the liver in humans [41].

However it should be taken into consideration that there is no unique ‘gold standard’ in diagnosis of AE, only a combination of clinical trials, imaging and laboratory diagnostic techniques can allow to diagnose a confirmed, probable or possible invasion.

The detection rate for human AE among people who live in the endemic areas in the northern part of the Varmia – Masuria Province (Table 2) was very high, in particular in the communities of Srokowo, Barciany, Budry and Kiwity. According to Eckert and Deplazes [9], the annual incidence of human AE in Europe varies from 0.01–0.3/100.000 inhabitants depending on the country. A local annual incidence of two or more AE cases per 100 000 is regarded as a high endemic area. As can be seen in Table 2 this criterion of high endemicity was fulfilled for 4 communities of the Varmia – Masuria Province. These communities are located in the formerly German (until 1945) East Prussia (Ost – Preussen) territory, whereas now three of them border the Russian Kaliningrad enclave. Fife AE cases (including 5 affecting members of a family) were recorded in the Srokowo community with a population of 4293 inhabitants. This is a sparsely populated, marshy, woodland, with a high population of the red fox (*Vulpes vulpes*) and raccoons dogs (*Nycterentes procyonoides*). Furthermore in two other communities the incidence was 1.97/100 000. It should be emphasized that the average detection rate for human AE in the Varmia – Masuria Province in the years 1990–2011 was 0.20 as compared with 0.014 per 100 000 inhabitants in all the country (Table 2).

Since the beginning of AE project it was decided that one experienced surgery center should be selected in a medical university. In fact there were two surgical clinics in Medical University of Warsaw. There were a total of 35 surgeries done including 12 OLTs.

Thus, out of a total of 725 liver transplantations performed in CDGTLS MUW from January 1989 to December 2008, 11 livers were transplanted because of AE (6.4%), the main indication being parasitic bile duct infiltration and resultant cholestasis (in the years 1990–2011 were 1034 transplantations in this 14 with AE). This percentage is over twelvefold higher than in the material of European Liver Transplantation Registry, presented in 2005, in which among 79 044 LT cases, parasitic disease (not only AE) was reported in 3.0% of the cases [29]. In our material, cumulative 1, 5 and 10 year recurrence – free survival rates after OLT in the alveolar echinococcosis group of patients were: 100%, 91% and 74.3% respectively [29]. Apart from few indications, liver transplantation is abandoned in favour of major resections and palliative, sometimes multistage surgery. An aggressive surgical management involving removal of the possibly largest mass of the parasite provides beneficial conditions for chemotherapy and prolongs survival in advanced AE cases [35].

The results indicate the need of a wider national programme for implementation of screening (US, seroepidemiology) in the highest AE risk areas (north-eastern Poland) and increase of the public awareness of the possibility of contracting *E. multilocularis* and its consequences, and above all, training of the primary care physicians in the recognition of the risk of AE to allow for an early detection of this dangerous disease. This is consistent with the recommendations developed at the Swiss International Exploratory Workshop “Alveolar Echinococcosis in Poland, Lithuania and Switzerland”. Zurich, 2010, 17–19.11 (unpublished report).

In conclusion the epidemiological situation in Poland demonstrated that AE is an emerging diseases in this country.

### List of accession numbers for genes mentioned in the text

#### *Echinococcus multilocularis* mitochondrial DNA, complete genome

GenBank: AB018440.2

#### *Echinococcus multilocularis* mitochondrial gene for 12S rRNA, partial sequence

GenBank: AB031351.1

## Supporting Information

Checklist S1 **STROBE checklist.**
(DOC)
